# Supplementary material for: Refining the Y chromosome phylogeny with southern African sequences
Source: Hum Genet. 2016 Apr 4;135(5):541–53. doi: 10.1007/s00439-016-1651-0 (PMC4835522; doi:10.1007/s00439-016-1651-0)
Supplement: Supplementary file 1 [file 439_2016_1651_MOESM1_ESM.pdf]

Refining the Y chromosome phylogeny with southern African sequences

Human Genetics

Chiara Barbieri*, Alexander Hübner, Enrico Macholdt, Shengyu Ni, Sebastian Lippold, Roland Schröder, Sununguko Wata Mpoloka, Josephine Purps, Lutz Roewer, Mark Stoneking, and Brigitte Pakendorf **

* Corresponding Author: Chiara Barbieri, Max Planck Institute for the Science of Human History, Kahlaische Strasse 10 D-07745 Jena, Germany – barbieri.chiara@gmail.com - Phone: +49 3641 686 801 - Fax: +49 3641 686 868

** Corresponding Author: Brigitte Pakendorf, Dynamique du Langage, UMR5596, CNRS & Université Lyon 2, 14 avenue Berthelot 69363 LYON CEDEX 07, France - brigitte.pakendorf@cnrs.fr - Phone: +33 4 72 72 64 32 – Fax: +33 04 72 72 65 90

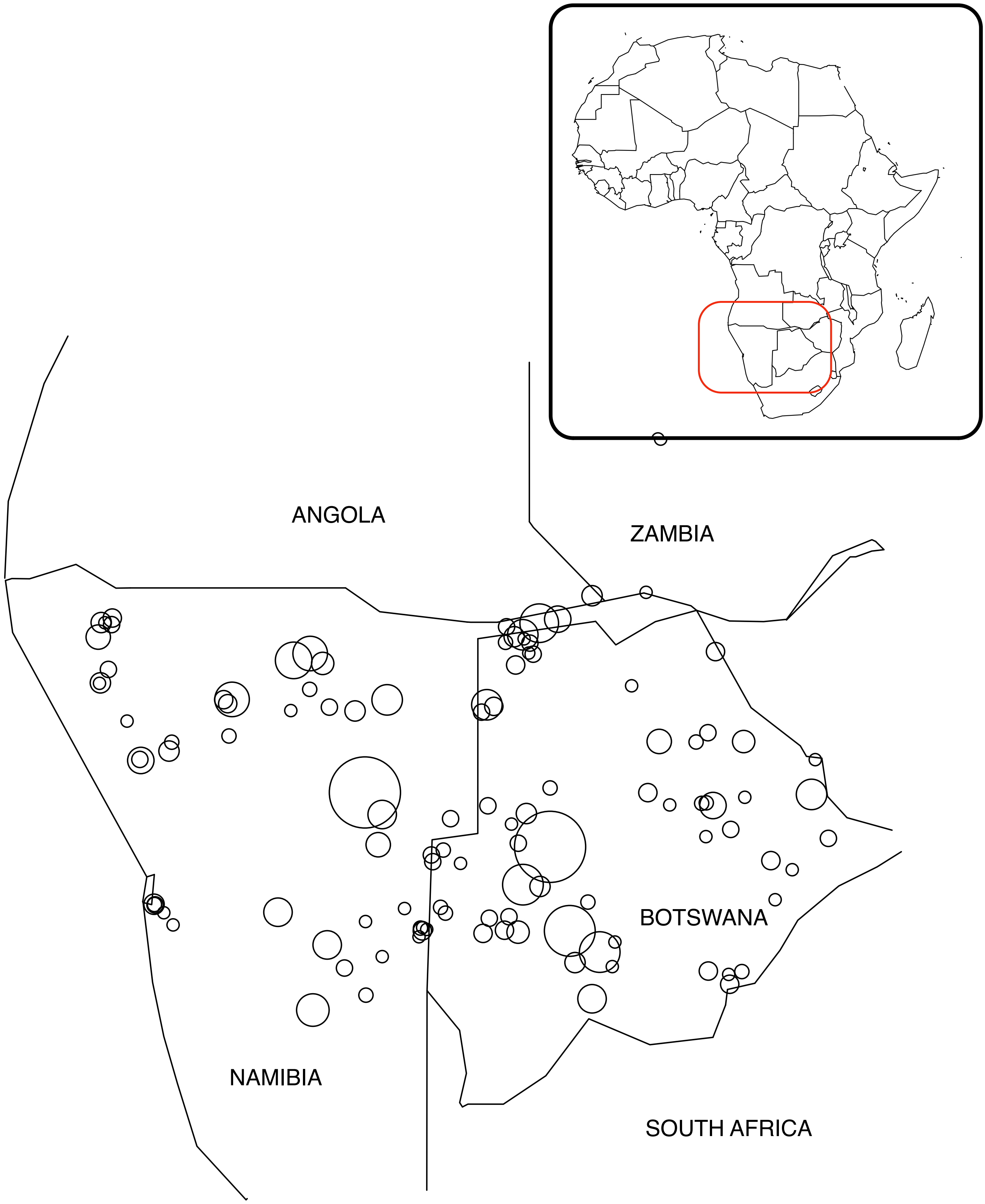

Figure S1 - Approximate sampling locations.
Circle size is proportional to number of individuals (minimum=1, maximum=30).

a

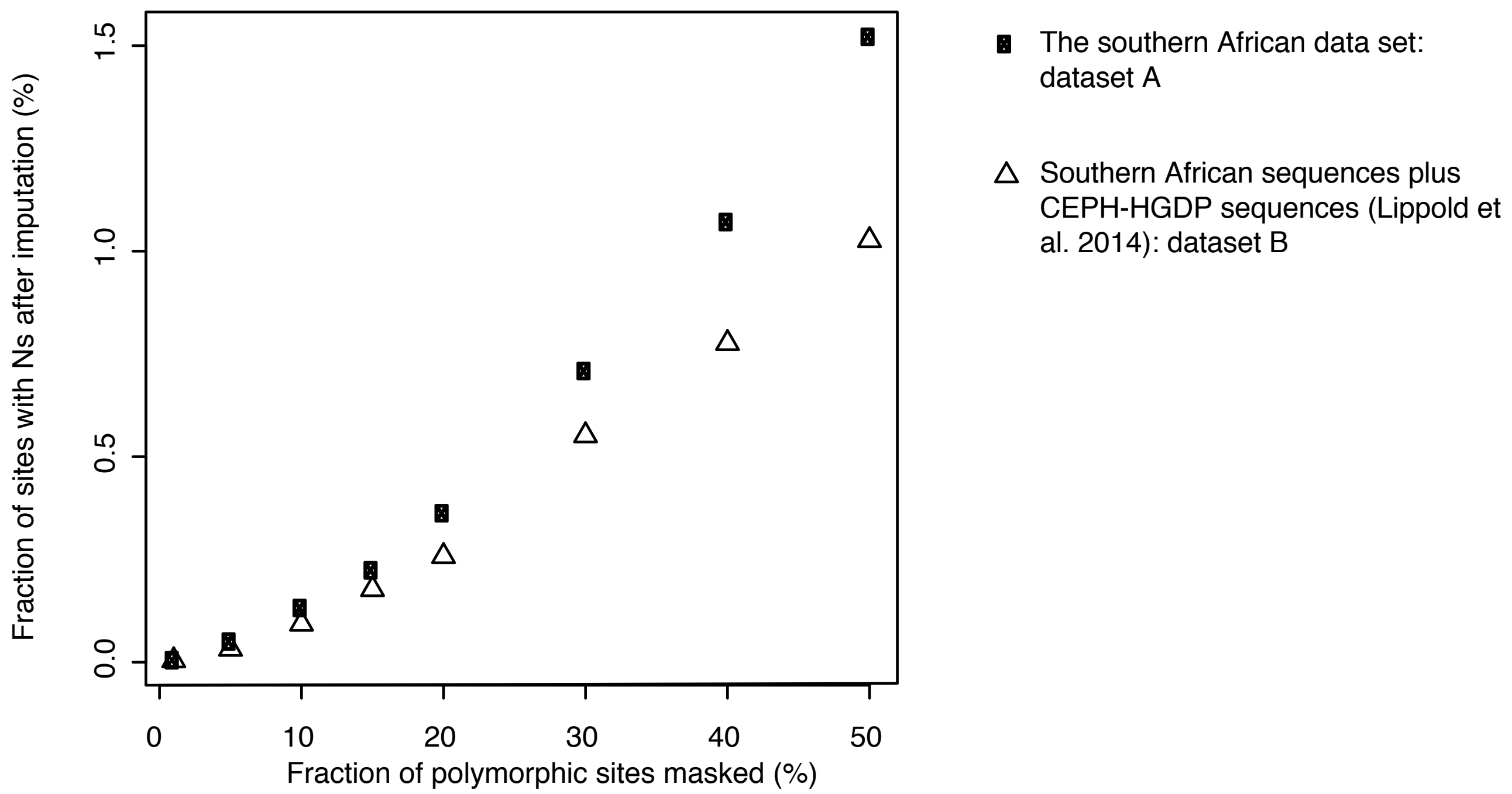

b

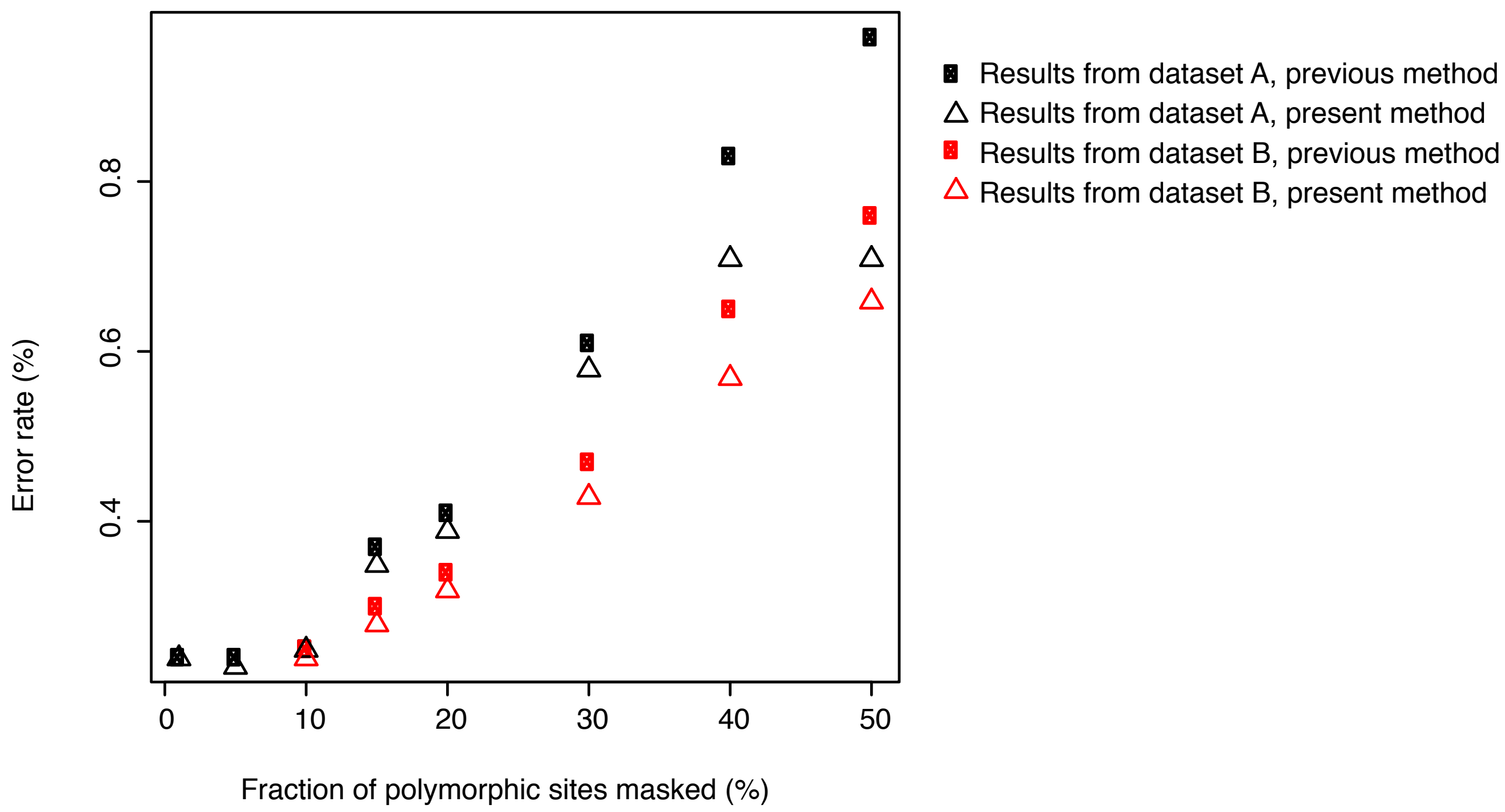

Figure S2 - Imputation performance.
a. Fraction of Ns left after imputation for the southern African sequences (dataset A) and the southern African sequences plus the sequences from Lippold et al. 2014 (dataset B); b. Comparison of the error rates for the imputation method used here and the method used previously.

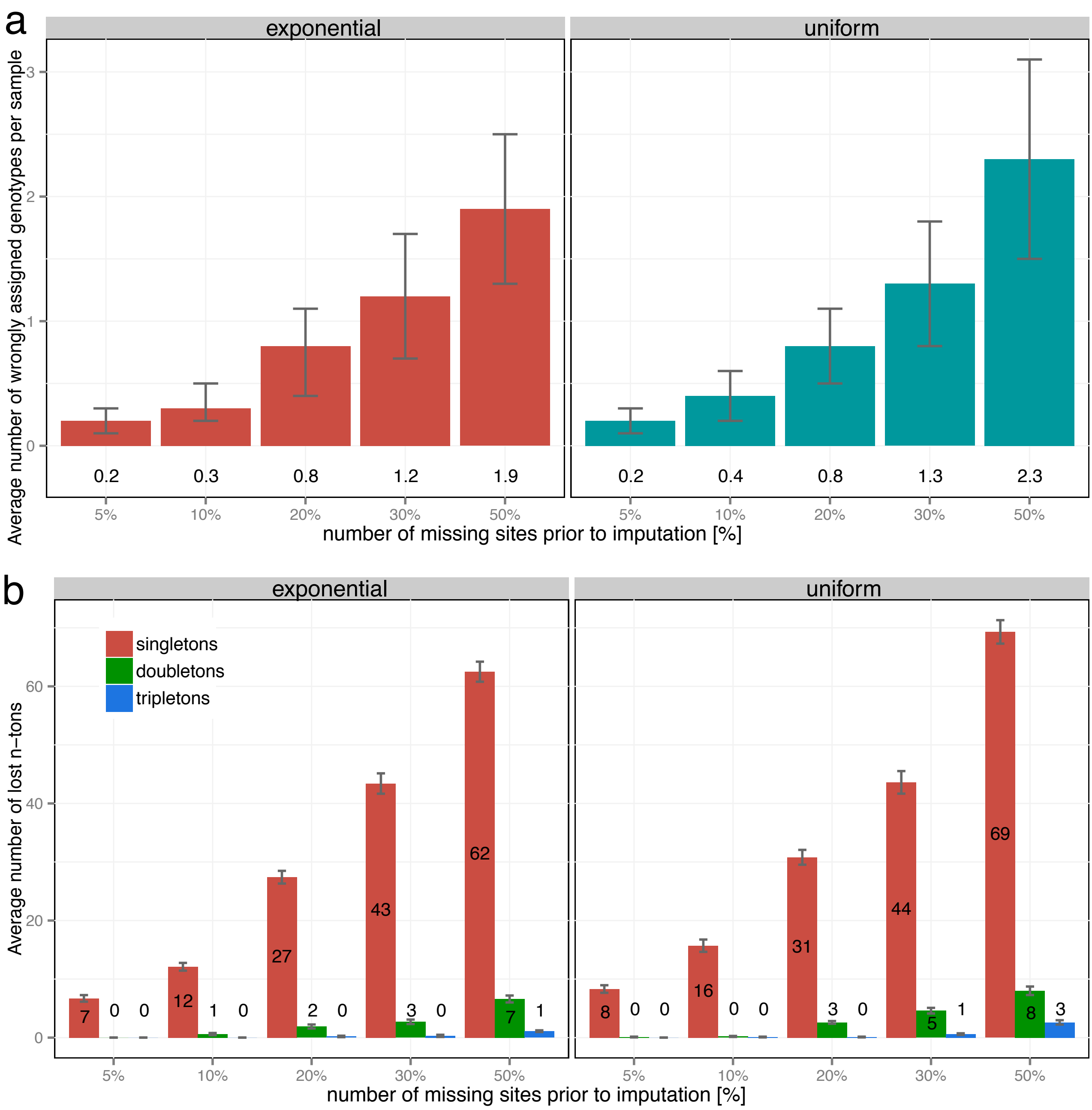

Figure S3 - Effect of imputation on observed genetic diversity.
a. Average number of wrongly-assigned genotypes for varying amounts of missing data prior to imputation, with sites with missing data distributed under either an exponential (left) or a uniform (right) model. The numbers under each bar indicate the average number of wrongly-assigned genotypes and the error bar is the 95% CI. The average number of wrongly assigned genotypes per sample was 0 for an upper boundary ≤ 10% and increased to up to 2 (i.e. 0.1%) for higher boundaries independently of the underlying sampling distribution. b. Number of singletons, doubletons and tripletons that were converted to invariant sites during imputation.

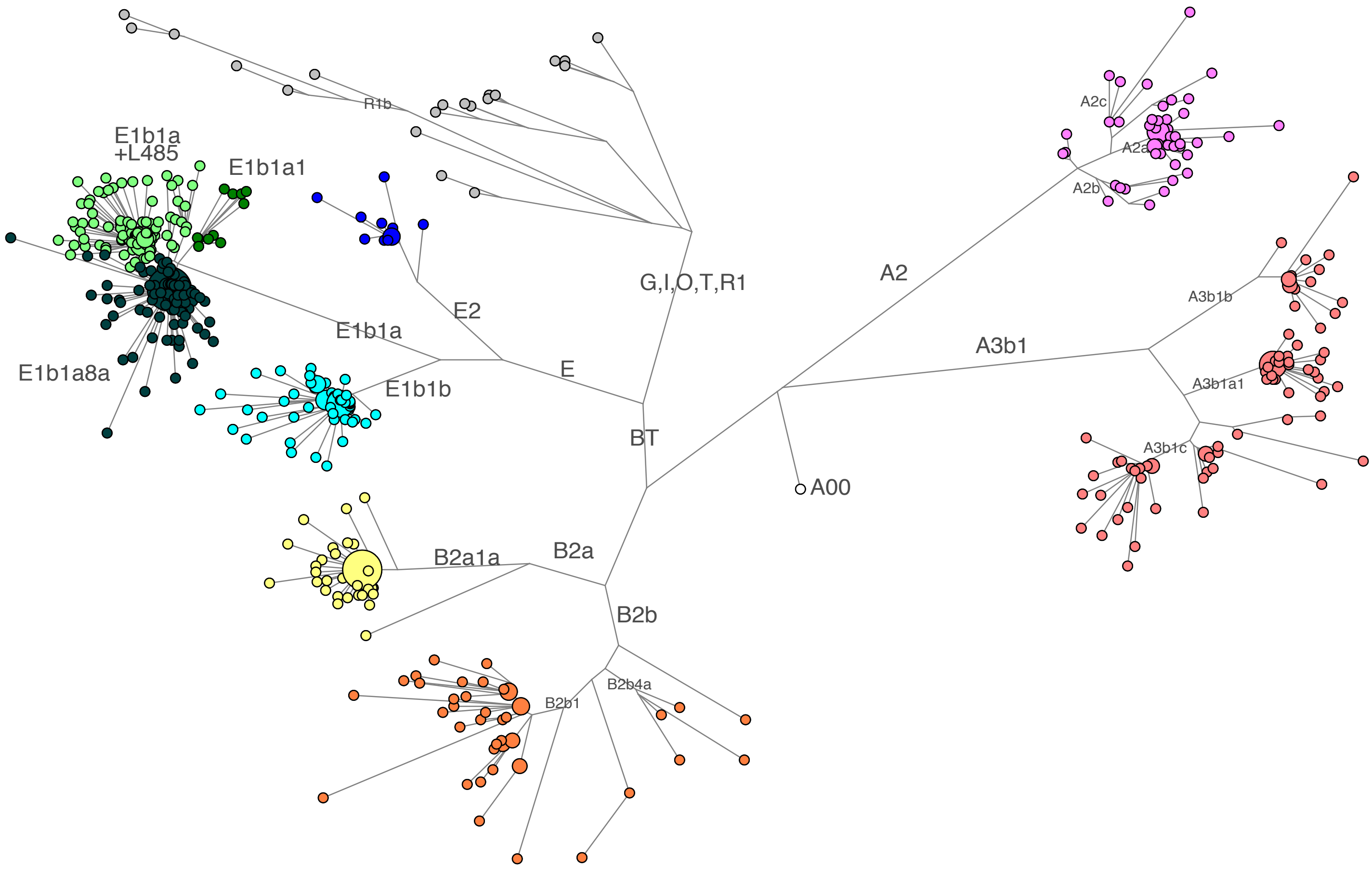

Figure S4 - Network of all of the sequences.

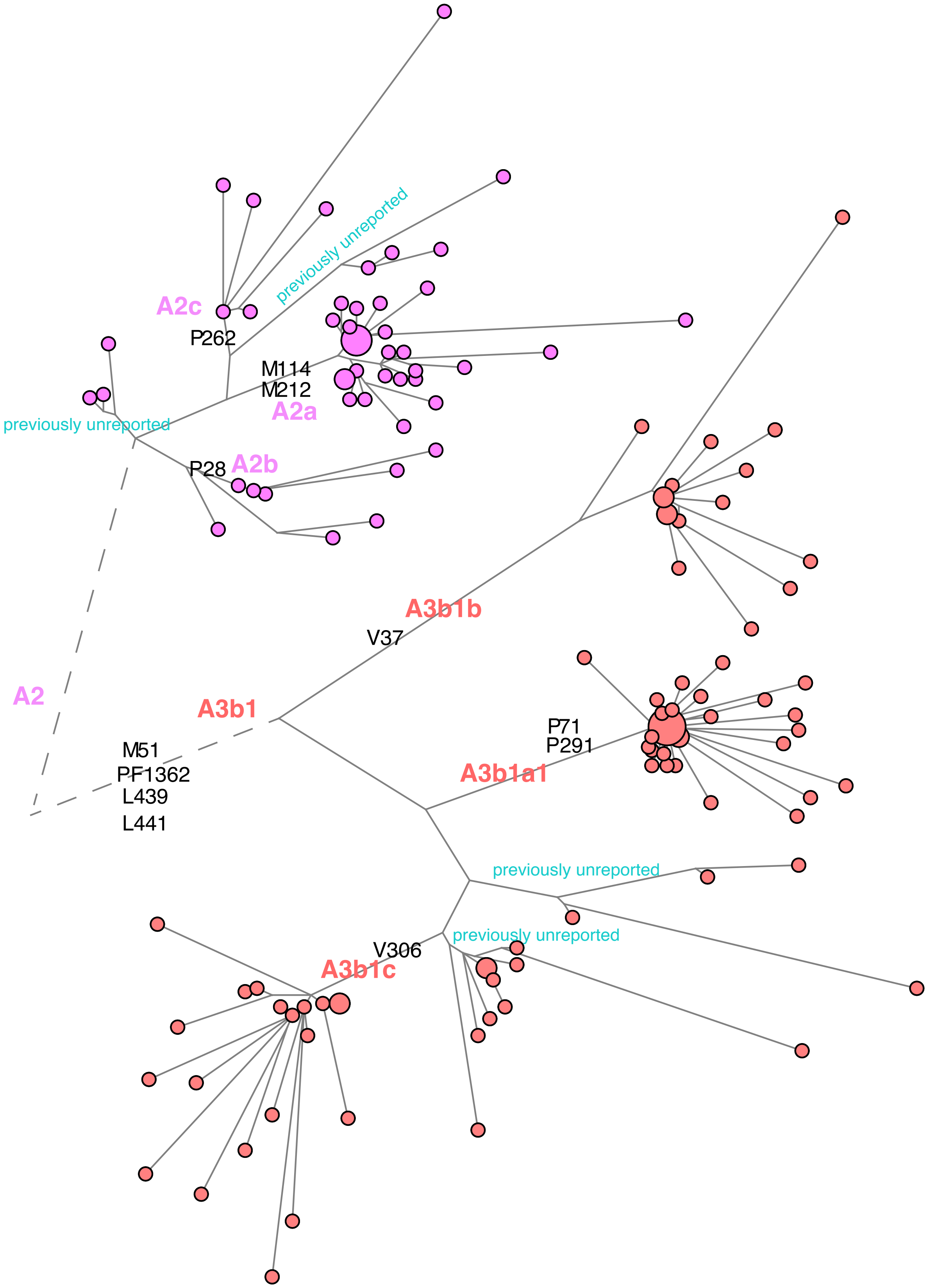

Figure S5 - Network of haplogroup A2 and A3b1 sequences (zoomed in from Figure S4).
Dashed lines indicate branches shortened for graphic purposes. Mutations reported in ISOGG and covered both by our sequences and by the additional genotyping are indicated on the corresponding branches.

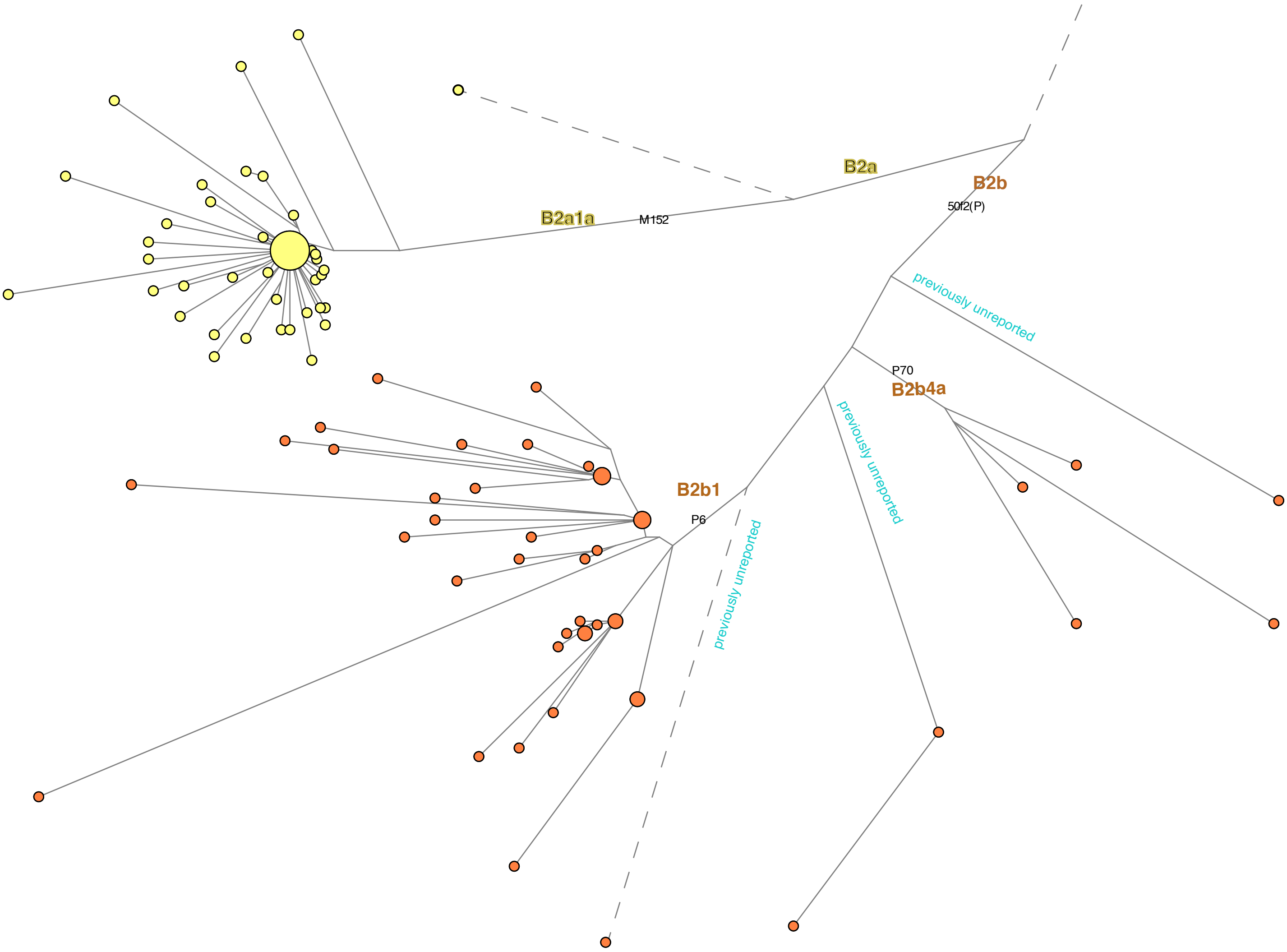

Figure S6 - Network of haplogroup B2a and B2b sequences (zoomed in from Figure S4).
Dashed lines indicate branches shortened for graphic purposes. Mutations reported in ISOGG and covered both by our sequences and by the additional genotyping are indicated on the corresponding branches. Note that the branch labeled as "B2a" is not confirmed by any diagnostic SNPs from ISOGG, but is assumed as the most plausible branch upstream from B2a1a.

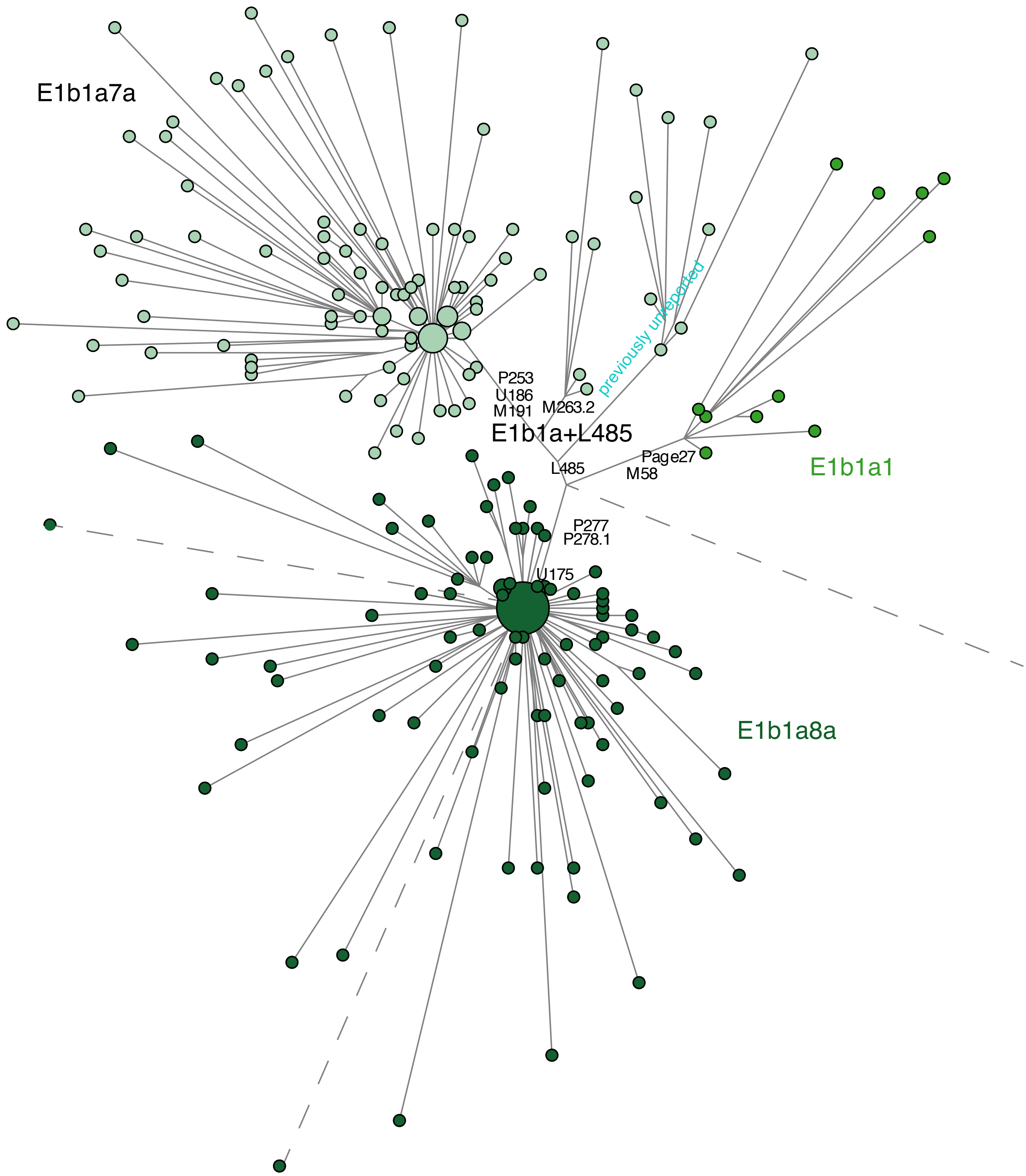

Figure S7 - Network of sequences from haplogroups E1b1a8a, E1b1a1, and E1b1a+L485 (zoomed in from Figure S4).

Dashed lines indicate branches shortened for graphic purposes. Mutations reported in ISOGG and covered both by our sequences and by the additional genotyping are indicated on the corresponding branches.

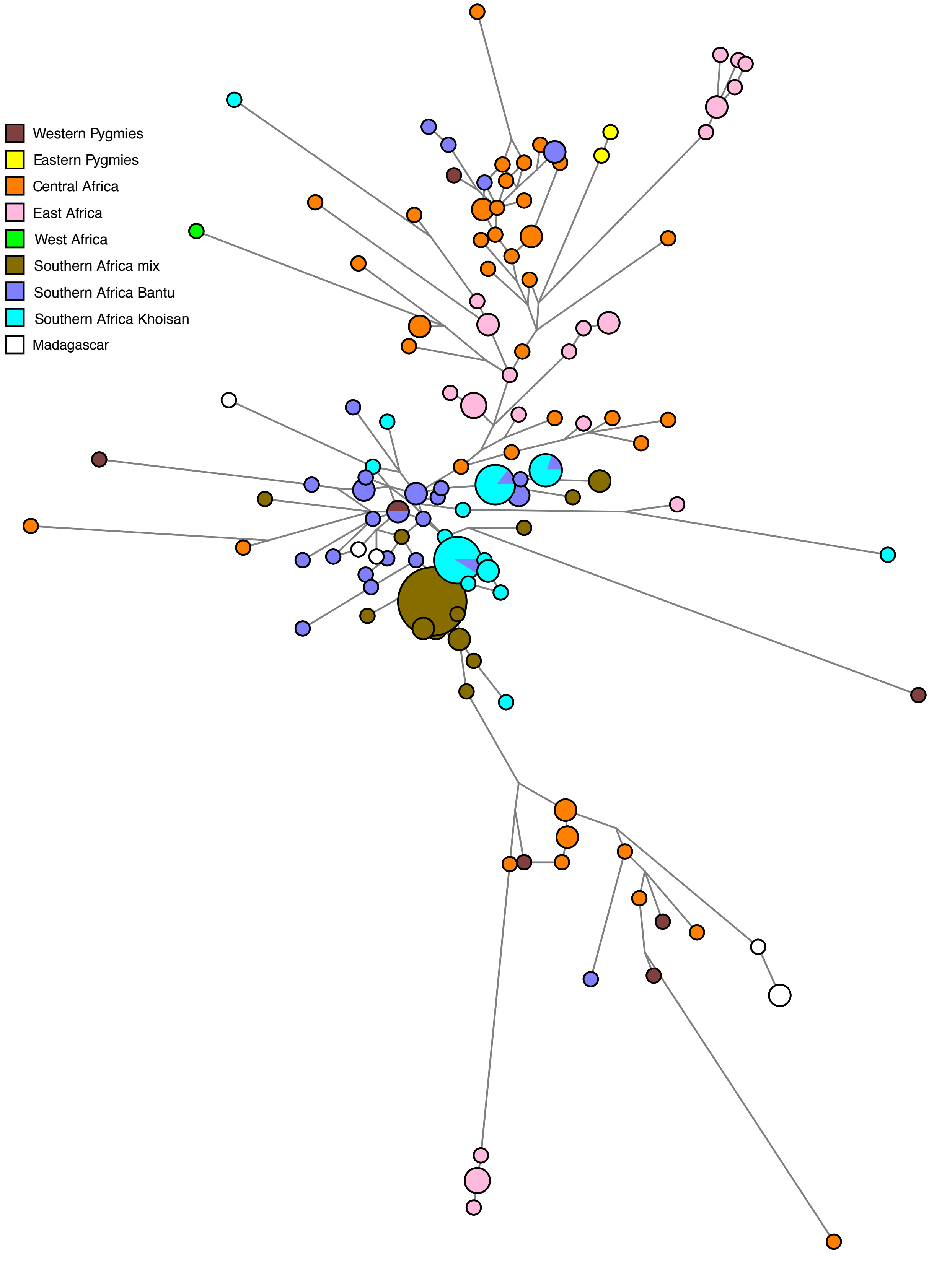

Figure S8 - Median Joining Network for B2a sequences, based on 16 STR loci and including published data (Batini et al. 2011).
The haplotypes are color coded to indicate major geographical areas and populations. The sample "Southern Africa mix" corresponds to a mixed sample of Bantu and Khoisan speakers (Batini et al. 2011). Haplotypes are available in Table S4.

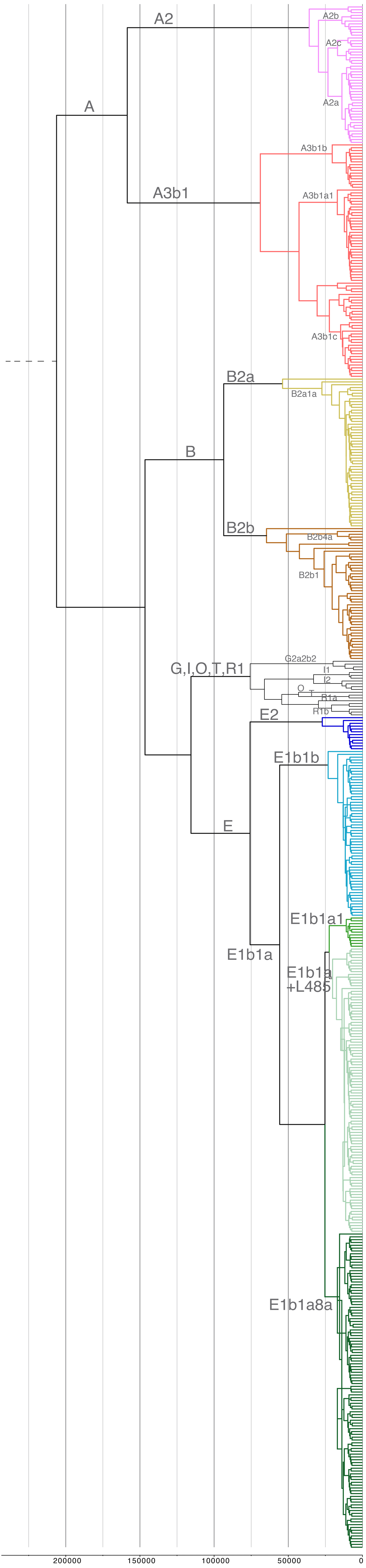

Figure S9 - Bayesian tree for the 547 southern African sequences.
Haplogroups are color-coded as in Figure 1.

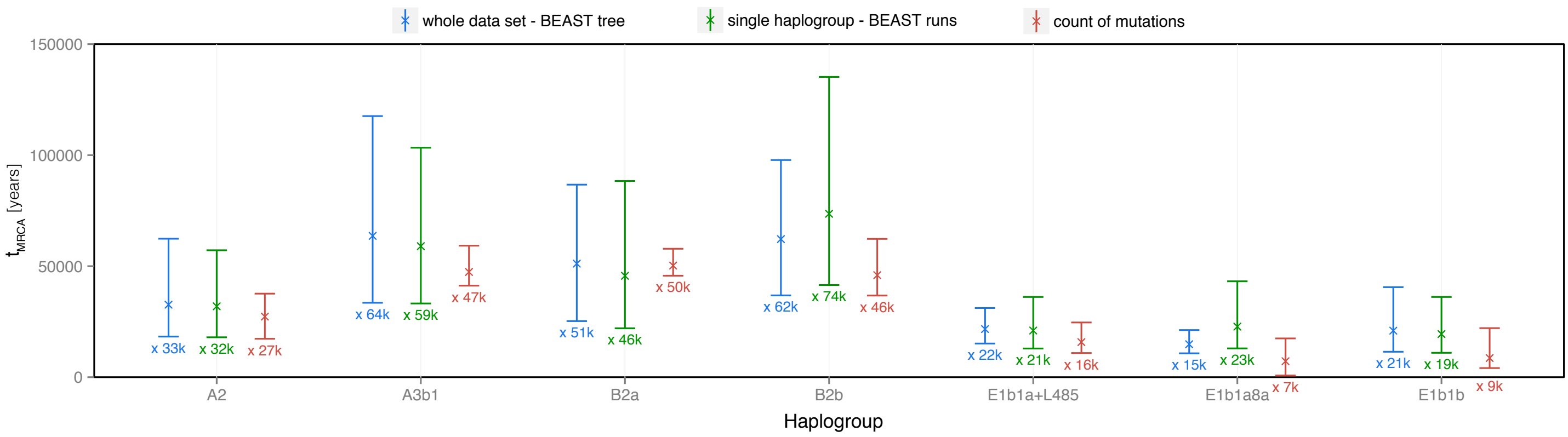

Figure S10 - Comparison of TMRCA estimates for the major haplogroups.
Based on: a BEAST run of the entire dataset (Figure S9); a BEAST run for the specific haplogroup; and from the count of mutations from the root.

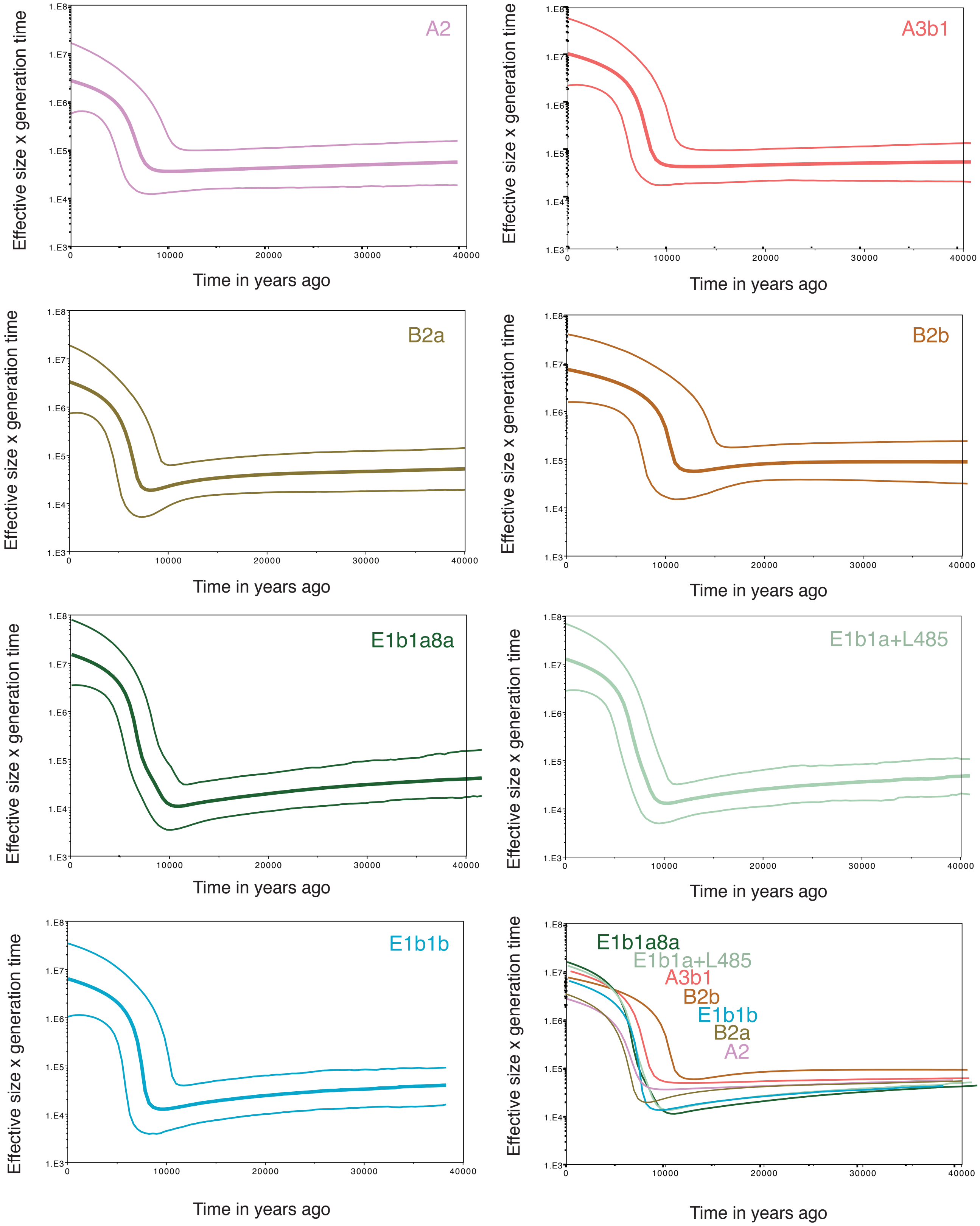

Figure S11 - BSP plots for the major haplogroups, with a relaxed exponential clock model and a mutation rate of 0.82x10^{-9}.
The thick line is the median estimate, bracketed by thin lines for the 95% HPD intervals. Bottom right, a summary BSP with the separate haplogroup median lines.

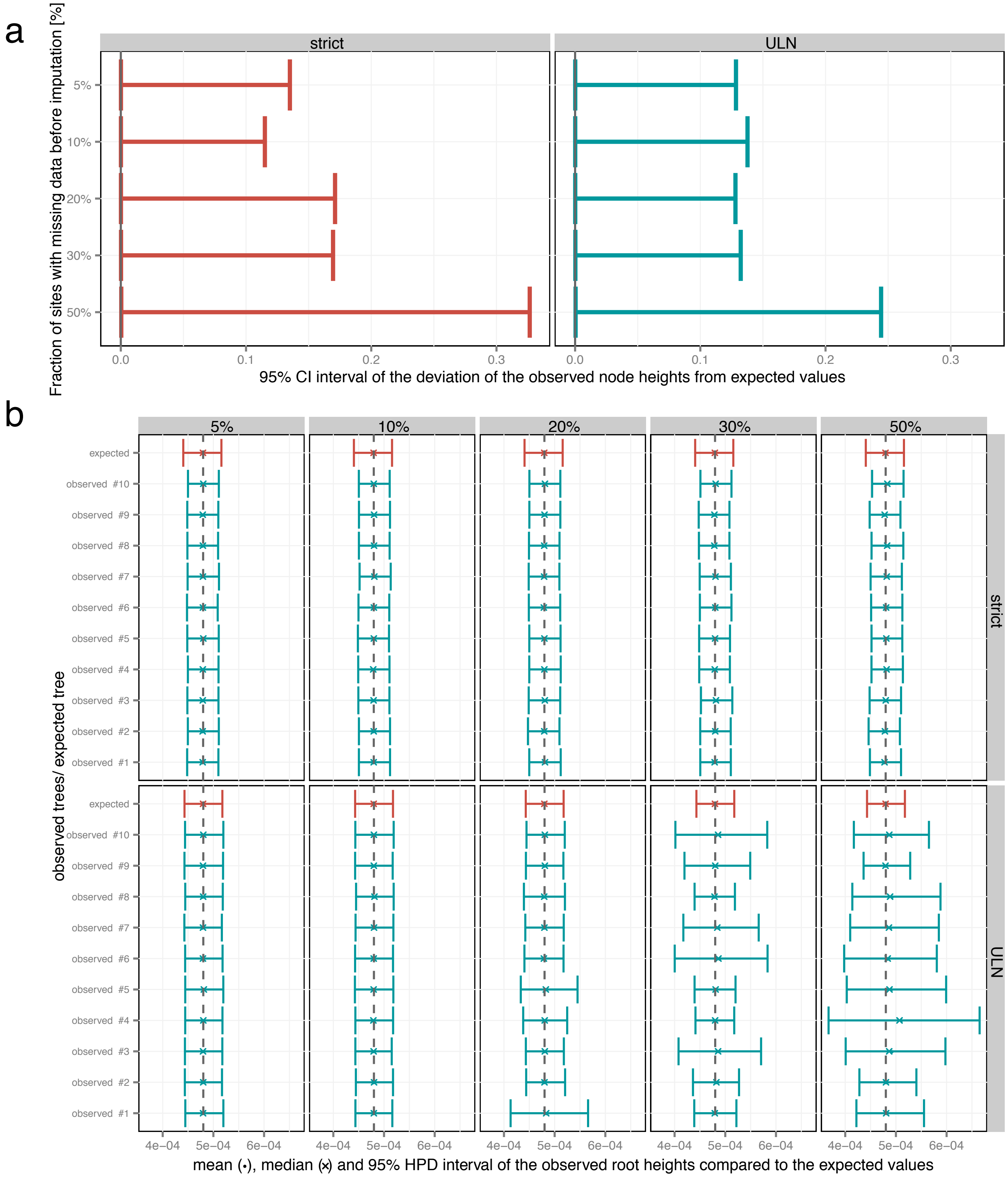

Figure S12 - Effect of imputation on the Bayesian phylogenetic analysis using BEAST.
a. Distribution of the sum of the squared deviation of all node heights of the simulated observed trees (based on varying amounts of missing data before imputation) compared to the expected tree (without missing data) for strict and ULN clock models.
b. The mean (point), the median (cross) and the 95% HPD interval of the observed tree root heights (blue) of the 10 repeats per set of conditions compared to the expected tree root height (red) calculated with a strict clock model (upper) and a ULN clock model (lower).

a

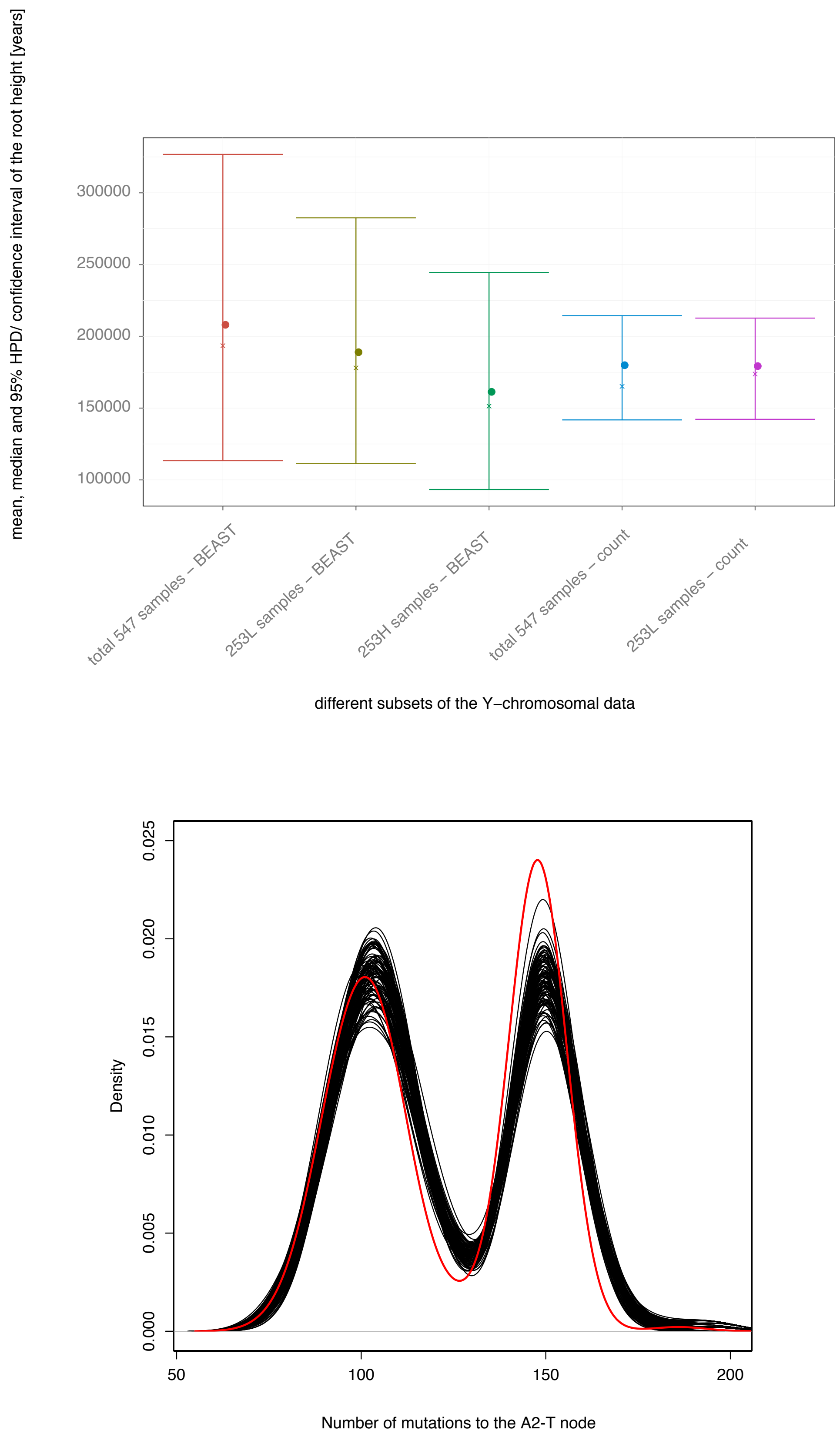

Figure S13 - Impact of imputation on the root height estimation in the southern African data and a less imputed dataset.
a. 95% HPD interval for the root height of BEAST MCC trees for the entire data set of 547 sequences, the less-imputed data set of 253 sequences (253L), and a random sample of 253 sequences (253H) from the entire data set. For the TMRCA estimates obtained by counting the mutations along the branches the 95% confidence interval is shown. The circle in each interval is the mean and the cross is the median. b. Distribution of the number of mutations to the A2-T node for the 253L (red) and 253H datasets (black).

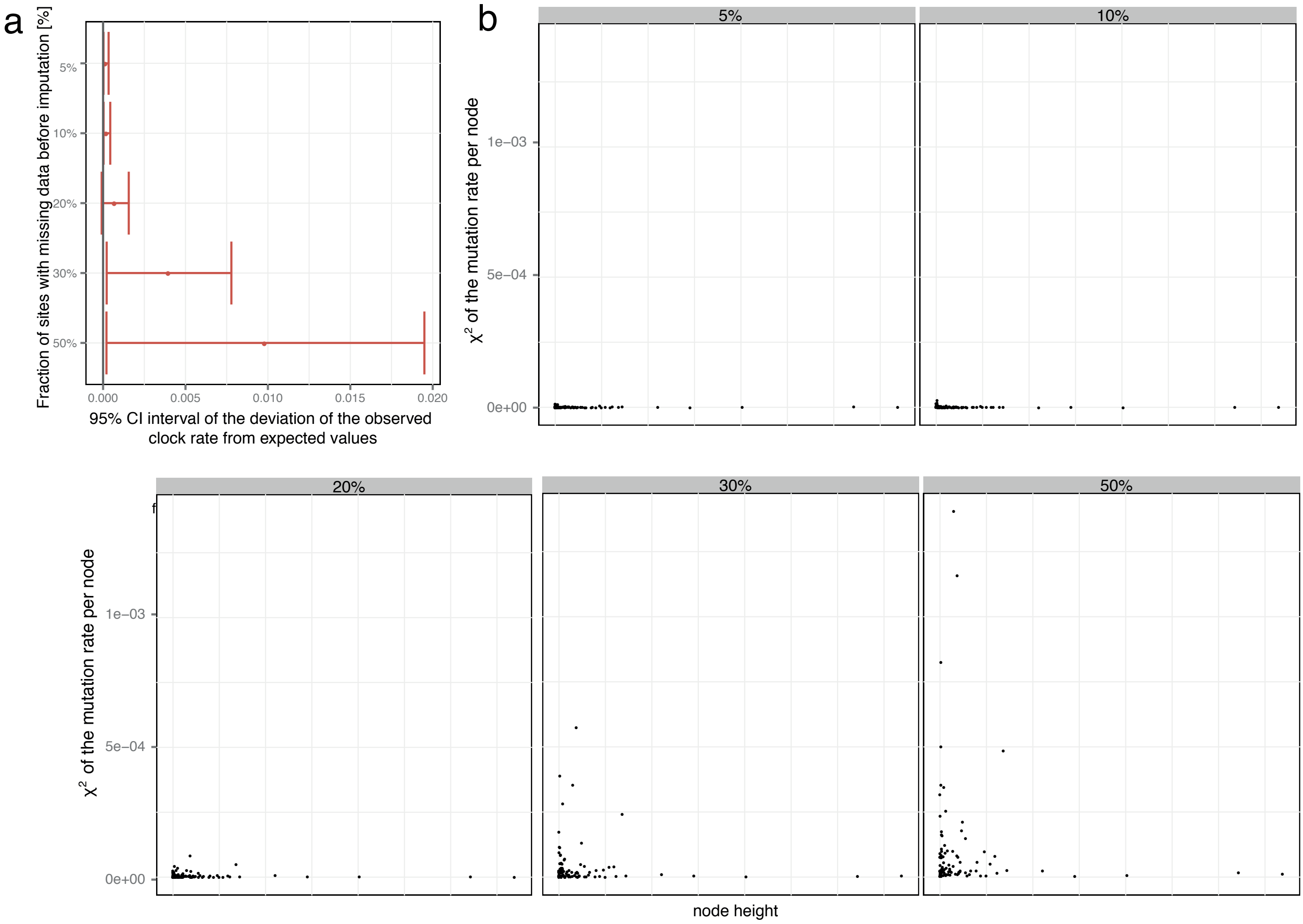

Figure S14 - Impact of imputation on variation in the clock rate across the tree for simulated data.
a. Distribution of the sum of the squared deviation of the clock rates of the simulated observed trees (based on varying amounts of missing data before imputation) compared to the expected tree (without missing data).
b. Scatter plots of the squared deviation values involving clock rates plotted against node height on the tree. Plots are shown for varying amounts of missing data prior to imputation.

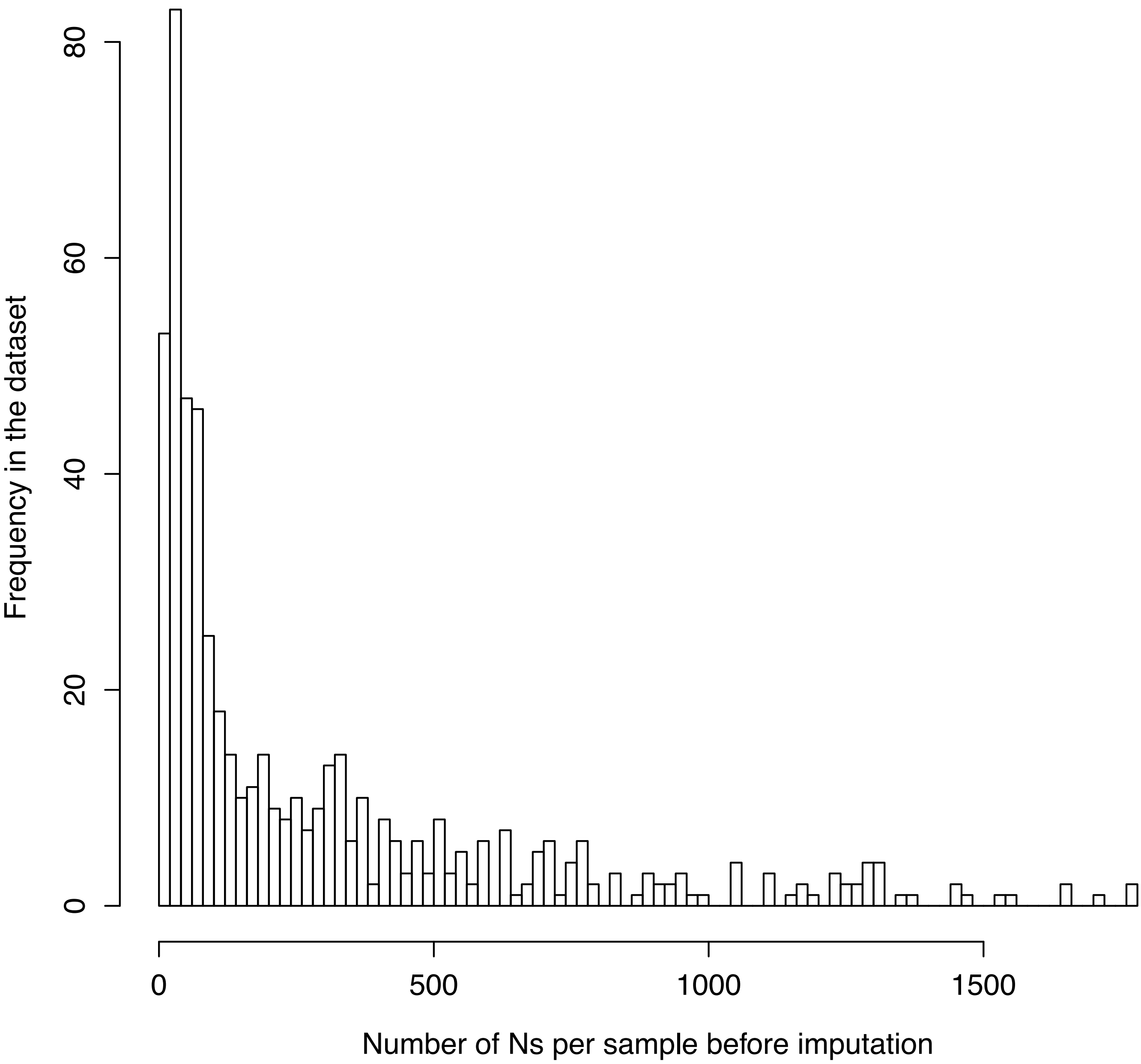

Figure S15 - Number of sites with missing information per sample before imputation.

a

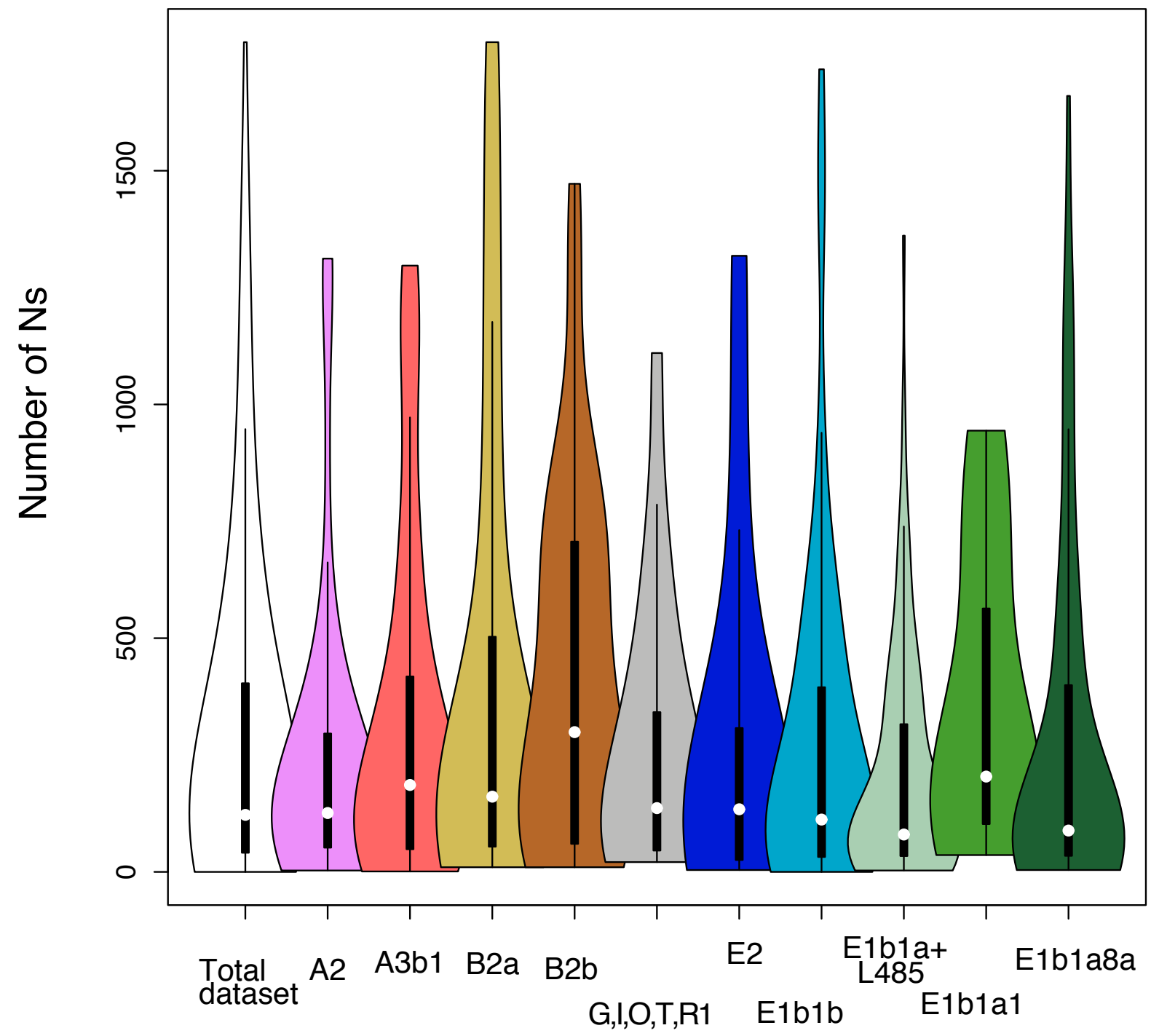

b

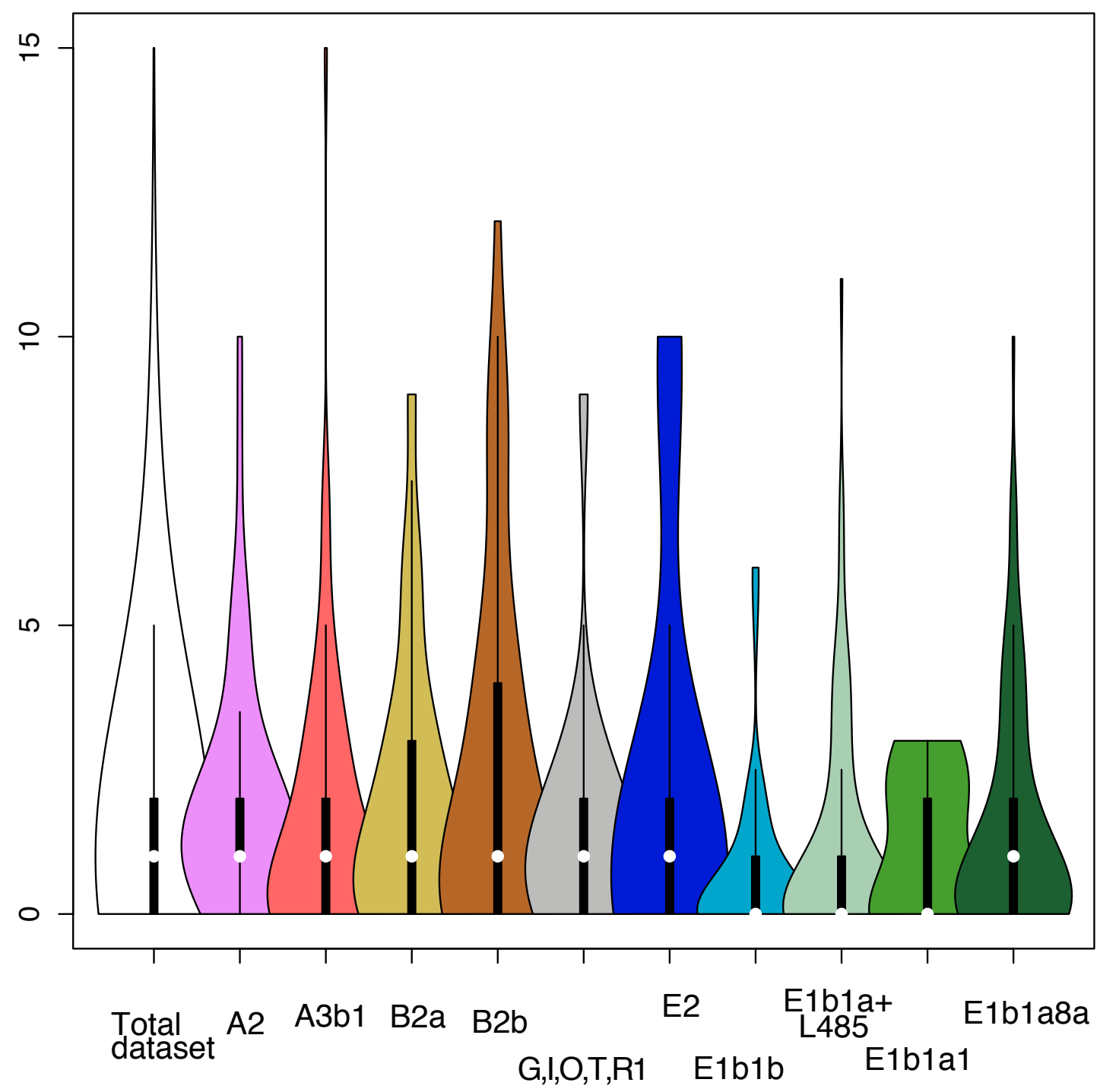

Figure S16 - Impact of imputation on the major haplogroup branches.
a. Distribution of the number of sites with missing information before imputation. b. Distribution of the number of sites with missing information after imputation.

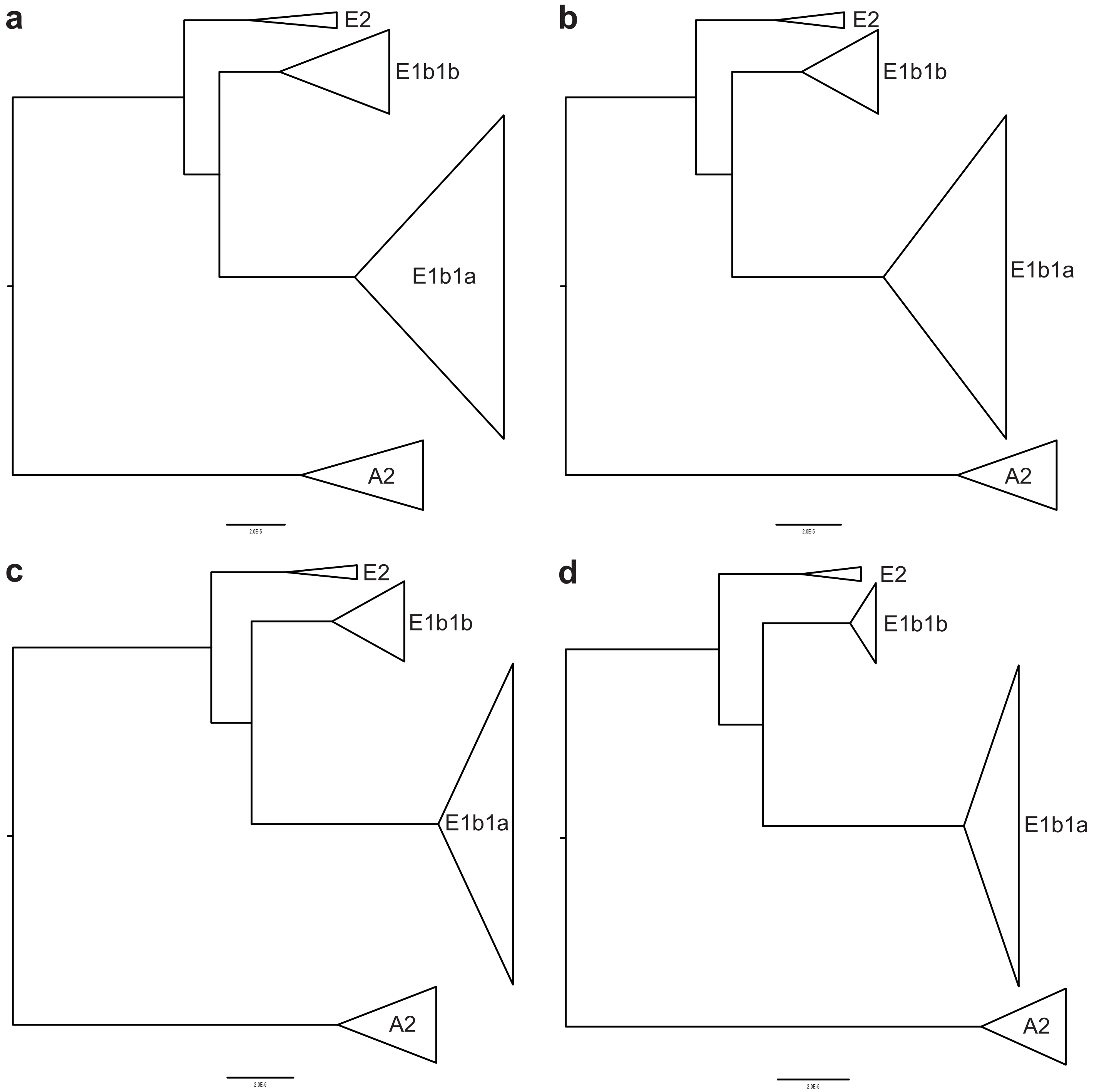

Figure S17 - Impact of sequence coverage on branch heterogeneity.
Maximum clade credibility trees of BEAST runs of all lineages of haplogroup E using haplogroup A2 as an outgroup. a. Data set based on 344 samples using 2,844 SNPs. b. Data set based on 344 samples using only sites for which the alternative allele was supported by at least three reads in at least one sample (2,028 SNPs). c. Data set based on samples with a minimum coverage of 10x in the target region (249 samples) and all sites (2,844 SNPs). d. Data set based the combination of the filter criteria of B and C (249 samples; 2,028 SNPs).
